# Book Reviews

**Published:** 1896-11

**Authors:** 


### Book Reviews.

TWENTIETH CENTURY PRACTICE. An International Encyclopedia of Modern Medical Science. By leading authorities of Europe and America. Edited by THOMAS L. STEADMAN, M. D., New York City. In twenty volumes. Volume VIII. Diseases of the Digestive Organs. New York: William Wood & Company. 1896.

The contributors to the volume before us are Dr. B. Farquhar Curtis, Dr. Max Einhorn, of New York; Dr. Reginald H. Fitz, of Boston; Dr. Joseph M. French, of Cincinnati; Dr. J. Ch. Huber, of Memmingen, Bavaria; Dr. Werner Kümmel, Dr. Johann Mikulicz, of Breslau; and Dr. Hans Leo, of Bonn.

The first section, written conjointly by Mikulicz and Kümmel, is devoted to a consideration of diseases of the mouth. This is a subject that has received little attention heretofore, and this chapter is deserving of careful examination in the light of newer pathology and advanced therapy. The second section relates to diseases of the esophagus, and is written by Fitz. This author is always painstaking, and has briefly set forth the salient features of diseases of this organ as at present understood.

The next section treats of diseases of the stomach, is written by Einhorn, and comprises about 240 pages of the book. As might be expected from the nature of this important disease, the subject is exhaustively handled by the distinguished author and contains many suggestions on pathology and treatment that are of quite recent origin ; indeed, it is the latest extended publication on the subject and contains its important bibliography to date. Credit is given to Drs. Charles G. Stockton and Allen A. Jones, of Buffalo, for work they have done and published in this department of medicine. Cancer of the stomach is always a subject of deepest interest and is well handled in this work. There is no department of medicine that demands closer study by the general practiser of medicine than diseases of the alimentary tract, and especially those of the stomach. It is here that the foundation is often laid for future diseases, that, neglected, may become uncontrolable ; or checked, may become easily curable. We cannot commend this treatise of Einhorn too highly.

The next section takes up diseases of the pancreas and is by Leo. Here our knowledge is limited, hence the author writes briefly upon his chosen subject ; nevertheless, what he presents may be regarded as an epitomé of our knowledge of the diseases of the pancreas up to the present time. The following section is devoted to diseases of the peritoneum and is by Curtis. This author devotes about 100 pages of the volume to the consideration of his subject, which is more or less exhaustive in character. Under special forms of peritonitis he includes appendicitis, which is a subject very much in evidence at present. About forty pages of this section are taken up with this subdivision, which is an exceedingly interesting resumé of our present knowledge of appendicitis. Curtis' entire chapter is of great value and cannot help but aid the general physician in the solution of many difficult problems that are presented to him at the bedside.

The next section discusses animal parasites and the diseases caused by them, and is written by Huber. Of special interest in this chapter will be found a dissertation on the development of echinococcus cysts. Huber is also again interesting in his discussion of filaria and trichina. The final section of the volume is devoted to the consideration of the treatment of diseases caused by animal parasites, and is written by French. A comprehensive index finishes the volume.

This series is readily assuming a definite and fixed place in the literature of medicine and will be found a valuable supplement to even an already overgrown library. If, however, there has been in the minds of some a question as to the propriety of occasionally issuing cyclopedic systems of medicine, there can now no longer be any question as to the appropriateness of publishing this one. It is demonstrating its value and importance volume by volume.

The present volume has been issued out of its order, hence the next to be published will be Volume VII.

---

A Compend of Diseases of Children. Especially adapted for the use of Medical Students. By Marcus P. Hatfield, A. M., M. D., Professor of Diseases of Children, N. W. U. Medical School, etc. Quiz Compends, No. 14. Duodecimo, second edition, thoroughly revised. Price, 80 cents. Philadelphia: P. Blakiston, Son & Co., 1012 Walnut street. 1896.

If the quiz compend were used properly as a handy dictionary of diseases, or terms in a special branch of medicine, it would not be so objectionable. The student who depends on it for more is certain to have superficial knowledge where he should be well grounded. Especially is this true in pediatrics. Still, for a work of this kind, the book is a good one, except in a few particulars. It is not up to date on the subject of infant feeding, and we doubt if the paragraphs devoted to its consideration received the thorough revision given the rest of the volume.

---

The Diagnosis and Treatment of Diseases of the Rectum, being a Practical Treatise on Fistula, Piles, Fissure and Painful Ulcer, Procidentia, Polypus, Stricture, Cancer, and the like. By William Allingham, F. R. C. S., England; late Senior Surgeon to St. Mark's Hospital for Diseases of the Rectum, etc., and Herbert W. Allingham, F. R. C. S., England, Surgeon to the Great Northern Hospital, etc., 8vo, pp. xvi.—485. Sixth edition. New York: William Wood & Company. 1896.

This classic treatise is to be found on the shelves of every specialist in diseases of the rectum, and these, together with many physicians who do not class themselves as specialists, will be glad to obtain a new and revised edition. The two Allinghams—father and son—have long been considered authority on this subject. Mr. Allingham, Sr., was the first to put forth a distinct treatise on diseases of the rectum, and his labors in this field have served to create this line of practice into a separate specialty.

Previous editions of this work have become exhausted, hence it was out of print and could not be obtained by those who desired to consult it. The present edition is like the last, with such additions and changes as were necessary to bring it abreast of the period.

For clearness of expression this work is famous. No one, for instance, need be in doubt as to the nature of fistula-in-ano after reading Allingham's description of it, or how to cure it after studying his method. The drawings, too, seem to explain or elaborate the text with an exactitude that is most agreeable. How can one fail to understand the pathology and treatment of hemorrhoids after a careful examination of Allingham's illustration?

Another point to which we invite attention is the marginal references in this book. These make it possible to consult the text on a particular point in question without wading through pages irrelevant, thus economising time.

Finally, the paper and presswork are excellent, making it an agreeable book to read as well as consult. It will be a long time before Allingham's standard treatise will cease to be valued by physicians and students who desire to refresh their memories or to obtain ideas.

---

Diet for the Sick. Contributed by Miss E. Hibbard, Principal of Nurses' Training School, Grace Hospital, Detroit, and Mrs. Emma Drant, Matron of Michigan College of Medicine Hospital, Detroit. Second edition, enlarged. Limp cloth, 16mo, 100 pages. Price, 25 cents, postpaid. Detroit, Mich.: *The Illustrated Medical Journal Co.* 1896.

This little book may be found useful by the home nurse, whose ordinary cook-book contains few recipes for invalid dishes. It also contains quite complete diet lists and many suggestions which nurses, trained or untrained, may profit by. Its value is hardly increased, however, by the recommendation in the body of the work of proprietary articles of invalid food, and the physician will scarcely approve of the advice so freely given as to the employment of a certain new remedy in the treatment of dyspepsia.

